# B7-H7 (HHLA2) inhibits T-cell activation and proliferation in the presence of TCR and CD28 signaling

**DOI:** 10.1038/s41423-020-0361-7

**Published:** 2020-01-31

**Authors:** Sadiye Amcaoglu Rieder, Jingya Wang, Natalie White, Ariful Qadri, Catherine Menard, Geoffrey Stephens, Jodi L. Karnell, Christopher E. Rudd, Roland Kolbeck

**Affiliations:** 1 Biopharmaceuticals, Early RIA, AstraZeneca, Gaithersburg, MD USA; 2 Universite de Montreal, Montreal, QC Canada; 3 MedImmune/AstraZeneca, Gaithersburg, MD USA; 4 Present Address: Viela Bio, Gaithersburg, MD USA; 5 Present Address: Geneius Biotechnologies, Natick, MA USA

**Keywords:** T cell inhibition, Checkpoint pathway, T cell transcriptomics, Immunology, Adaptive immunity, Lymphocytes

## Abstract

Modulation of T-cell responses has played a key role in treating cancers and autoimmune diseases. Therefore, understanding how different receptors on T cells impact functional outcomes is crucial. The influence of B7-H7 (HHLA2) and CD28H (TMIGD2) on T-cell activation remains controversial. Here we examined global transcriptomic changes in human T cells induced by B7-H7. Stimulation through TCR with OKT3 and B7-H7 resulted in modest fold changes in the expression of select genes; however, these fold changes were significantly lower than those induced by OKT3 and B7-1 stimulation. The transcriptional changes induced by OKT3 and B7-H7 were insufficient to provide functional stimulation as measured by evaluating T-cell proliferation and cytokine production. Interestingly, B7-H7 was coinhibitory when simultaneously combined with TCR and CD28 stimulation. This inhibitory activity was comparable to that observed with PD-L1. Finally, in physiological assays using T cells and APCs, blockade of B7-H7 enhanced T-cell activation and proliferation, demonstrating that this ligand acts as a break signal. Our work defines that the transcriptomic changes induced by B7-H7 are insufficient to support full costimulation with TCR signaling and, instead, B7-H7 inhibits T-cell activation and proliferation in the presence of TCR and CD28 signaling.

## Introduction

Modulation of T-cell responses with biologics has been one of the greatest advances in medicine in the last decade.^1–3^ We now have a better understanding of T-cell biology and are able to fine-tune T-cell responses with different treatment options in cancer and autoimmunity.^4,5^ Some examples of treatment modalities include checkpoint inhibitors such as α-PD-L1 antibodies in cancer and a CTLA-4 Ig protein for the treatment of autoimmune conditions.^6,7^ In cancer and autoimmunity, T-cell activation is dysregulated in opposite ways. In cancer, there is a lack of T-cell infiltration and activation, whereas in autoimmunity T-cell activity is augmented. The process of T-cell activation is intricate, involves many different players, and is tightly controlled.^8^ Proper activation of T cells requires two important signals: signal 1 (the binding of T-cell receptor--TCR to peptide antigen-bound major histocompatibility complex, MHC) and signal 2 (the binding of CD28, a costimulatory molecule on T cells, with B7-1 (CD80) and B7-2 (CD86) on the antigen-presenting cell). In the context of both signals, T cells become activated, produce cytokines, and proliferate.^9^ Some reports demonstrate that T cells can also be activated, independent of those two signals, e.g., after cytokine receptor stimulation.^10^ The immune system also employs different mechanisms to control the hyperactivation of T cells, including the expression of coinhibitory molecules on the cell surface.^11^ The balance between costimulatory and coinhibitory molecules on T cells then determines the extent of T-cell responses in a host.

T cells express different costimulatory (i.e., CD28 and ICOS) and coinhibitory molecules (i.e., PD-1, CTLA-4, Tim-3, and LAG-3) on their surface, which generally belong to the B7 or TNF family.^12–14^ One of the most recently discovered B7 family ligands is B7-H7 (HHLA2) along with its receptor CD28H (TMIGD2 or IGPR1).^15,16^ B7-H7 is expressed on macrophages and activated dendritic cells, whereas CD28H is expressed on naive T cells, a subset of memory T cells, natural killer cells, Innate Lymphoid cells (ILCs) and plasmacytoid dendritic cells (pDCs).^15–18^ The role of this pathway is controversial. Initial studies demonstrated that B7-H7 inhibited cytokine production and proliferation by CD4 and CD8 T cells.^15^ In a separate paper, Zhu et al.^16^ found that this pathway increased T-cell growth and cytokine production in an Akt-dependent manner Since then, several publications have observed the coinhibitory role of this pathway in different cancer settings.^19,20^ In particular, B7-H7 is highly expressed in breast and lung cancers, as well as in osteosarcoma, and increased expression is associated with a poor prognosis.^21,22^ It is speculated that B7-H7 may be stimulatory or inhibitory depending on the receptor it binds, suggesting that in addition to CD28H, there may be an alternate receptor(s) for this ligand.^20^

In this study, we used B7-H7 in the form of a chimeric protein (the extracellular domain of B7-H7 fused to human IgG1 in the form of a disulfide-linked homodimer) for stimulation of human T cells. To gain mechanistic insights into B7-H7 stimulation, we performed RNA sequencing (RNAseq) and compared global transcriptomic changes induced by B7-1 or B7-H7. In combination with OKT3, B7-H7 induced a modest increase in the expression of select genes; however, the expression of many other T-cell activation-associated genes was not changed. Within the subset of genes that commonly exhibited upregulated expression induced by B7-1 and B7-H7, the fold change was significantly lower with the B7-H7 stimulation. In terms of functional outcomes, OKT3 and B7-H7 did not result in activation, cytokine production, or proliferation. This may be due to the induction of anergy, lack of ability to enter the cell cycle, and reduced pERK levels after B7-H7 treatment. In the context of OKT3 and B7-1, B7-H7 robustly inhibited T-cell activation, cytokine production and proliferation, and this treatment was comparable to PD-L1-induced inhibition. Furthermore, blocking the B7-H7 and CD28H interaction with a B7-H7-specific antibody increased proliferation in mixed lymphocyte reaction assays. Our work defines transcriptomic changes induced by B7-H7, points to a threshold for functional activation of T cells and suggests that B7-H7 inhibits T-cell activation and proliferation in the presence of TCR and CD28 signaling.

## Results

### B7-H7 induces fewer genes in select pathways than B7-1 and has no effect on some key activation genes

**Fig. 1:**
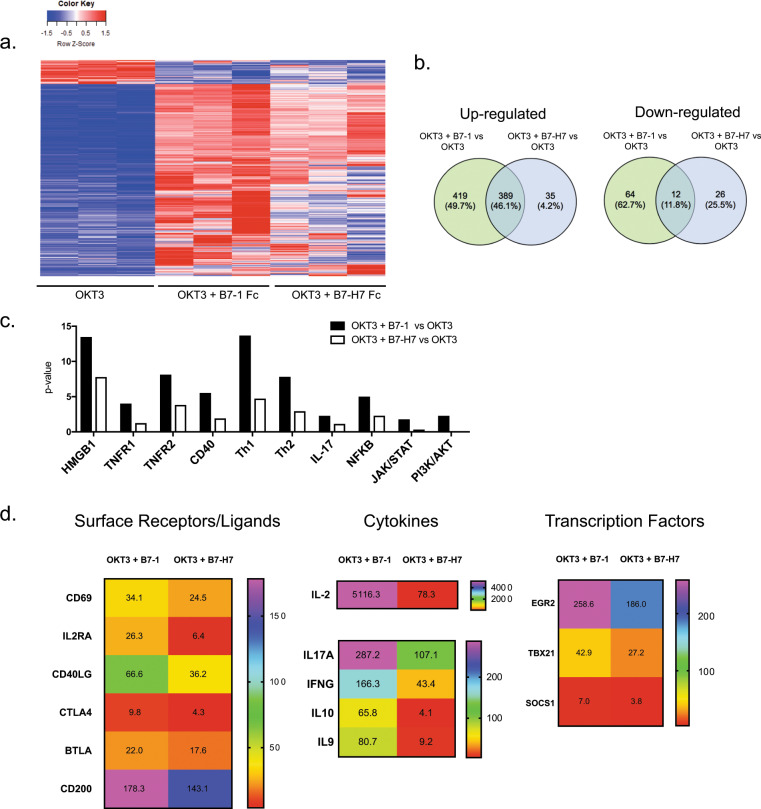
B7-H7 induces fewer genes in select pathways than B7-1 and has no effect on some key activation genes. Naive CD4 T cells were purified and stimulated with different treatment groups for 2 h. **a** Heatmap of significantly regulated genes in OKT3, OKT3 + B7-1 Fc, and OKT3 + B7-H7 Fc treatment groups. OKT3-treated cells were used as the reference in the generation of the heatmap. **b** Comparison of upregulated and downregulated genes between OKT3 + B7-1 Fc and OKT3 + B7-H7 Fc vs. OKT3 alone. **c** IPA pathway analysis showing differentially regulated pathways with OKT3 + B7-1 Fc and OKT3 + B7-H7 Fc stimulation compared with OKT3 alone. **d** Mean fold change expression of genes in OKT3 + B7-1 Fc, OKT3 + B7-H7 Fc vs. OKT3 alone, calculated from three different donors RNA-sequencing data. Representative genes in surface receptors, cytokines, and transcription factors are shown. For all RNA sequencing data fold change >4, FDR < 0.25 was used as a cutoff value (*n* = 3 donors, ***p ≤ 0.0001, ***p ≤ 0.001, **p ≤ 0.005, *p ≤ 0.05)

To understand the impact of B7-H7 on the global transcriptome in human T cells, RNAseq studies were performed. We purified naive CD4 T cells and stimulated them with plate-bound OKT3, OKT3 and Control Fc, OKT3 and B7-1 Fc, or OKT3 and B7-H7 Fc for 2, 4, or 24 h. Time-course experiments demonstrated that the optimal mRNA induction of select genes in response to B7-1 (*interleukin (IL)-2, interferon (IFN)-γ*, and *EGR1*) occurred at 2 h post stimulation (Supplementary Fig. 1a). Time kinetics were slightly different for OKT3 and B7-H7 stimulation in terms of IL-2 mRNA induction, as the peak was at 4 h. However, the fold change with B7-H7 stimulation was significantly lower than that with B7-1 stimulation. There were no differences in *IFNg* or *EGR1* mRNA levels when we compared B7-H7 stimulation at the 2 and 4 h time points. Therefore, 2 h time point was used for subsequent whole-transcriptome analysis. The overall gene expression profiles of naive T cells from three different donors after 2 h of stimulation with OKT3, OKT3, and B7-1 Fc, or OKT3 and B7-H7 Fc are presented in Fig. 1a. Cells treated with OKT3 alone were used as the reference sample and the heatmap was created with genes with a fold change > 4 and false discovery rate <0.25. All genes that fit these criteria were used to generate the heatmap and the common genes were not removed. Visual analysis of the heatmap showed significant differences in global gene expression between the different groups (blue: downregulated genes, red: upregulated genes). We also sequenced OKT3 and Ctrl Fc-treated T cells, and this treatment did not significantly change gene expression when compared with OKT3 stimulation alone, as the samples clustered very closely on the Principal Component Analysis (PCA) plot (Supplementary Fig. 1b). In total, 808 genes were upregulated and 76 genes were downregulated with OKT3 and B7-1 treatment, whereas 424 genes were upregulated and 38 genes were downregulated with OKT3 and B7-H7 treatment when compared with OKT3 stimulation alone (Fig. 1b). Of the upregulated genes, 389 were shared between the B7-1 and B7-H7 treatments, demonstrating that B7-H7 and B7-1 upregulate the expression of some common genes. None of the genes that exhibited upregulated expression induced by B7-1 displayed downregulated expression with B7-H7 treatment.

Pathway analysis with Ingenuity Pathway analysis software demonstrated that B7-H7 induced fewer genes in select pathways than B7-1 and these pathways included the HMGB1, TNFR1, TNFR2, CD40, Th1, Th2, IL-17, NFKB, JAK/STAT, and PI3K/AKT pathways. This means that B7-H7 activated fewer genes within these pathways, therefore resulting in a reduced *p*-value. For example, in the Th1 pathway, only 9 out of 20 genes exhibited significantly upregulated expression induced by B7-H7, and in the PI3K/AKT pathway only 2 out of 10 genes exhibited upregulated expression induced by B7-H7 (Fig. 1c). Further detailed analysis of select surface receptors, cytokines, and transcription factors showed that although B7-H7 resulted in modest upregulation of the expression of select genes, the level of fold induction was significantly lower than that induced by B7-1 (Fig. 1d). For example, *IL-2* expression was upregulated 5116.3-fold in OKT3 and B7-1 Fc-treated cells and 78.3-fold in OKT3 and B7-H7-treated cells compared with OKT3-treated cells.

### B7-H7 does not provide functional costimulation to T cells

**Fig. 2:**
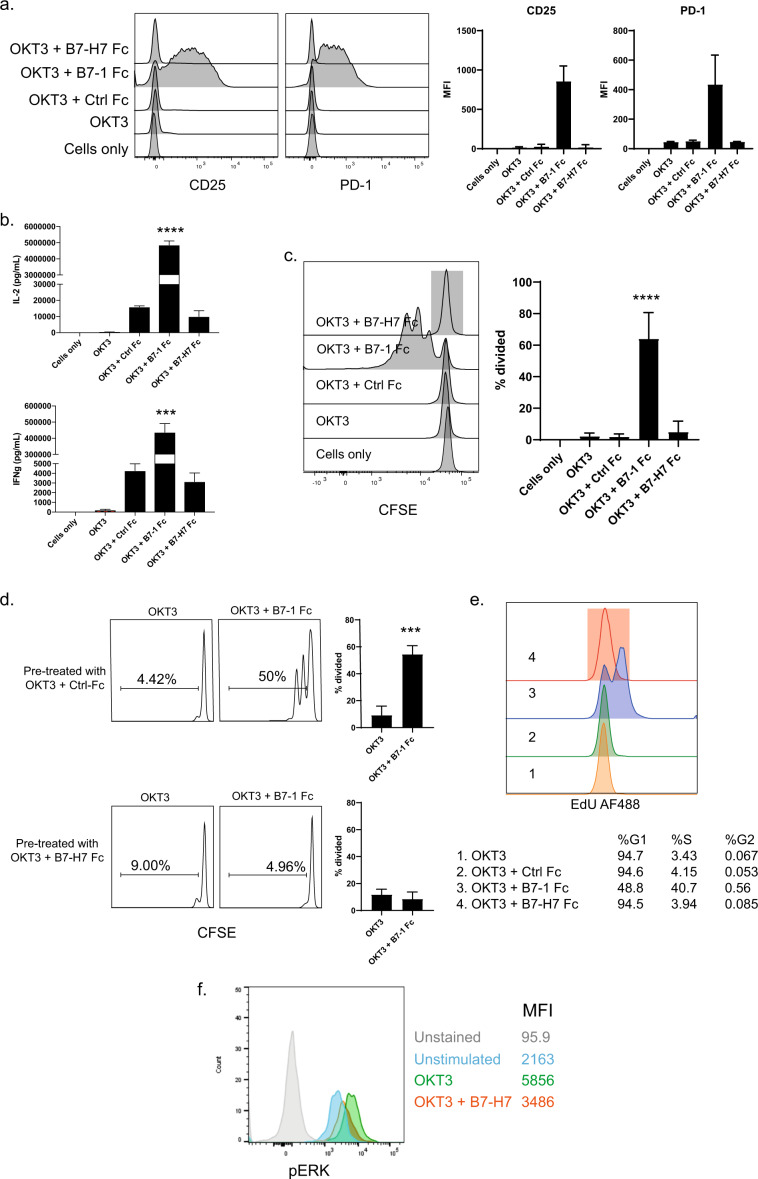
B7-H7 does not provide costimulation to T cells. Naive CD4 T cells were stimulated with OKT3, OKT3 + Ctrl Fc, OKT3 + B7-1 Fc, and OKT3 + B7-H7 Fc for 72 h. **a** The cells were then stained for CD25 and PD-1 protein expression. **b** IL-2 and IFNγ were measured in cell culture supernatants. **c** CFSE dilution was measured for cell proliferation. Bar plot summarizes data from three independent experiments. **d** Naive CD4 T cells were first pretreated with OKT3 + Ctrl Fc or OKT3 + B7-H7 Fc overnight, washed, and plated on wells coated with OKT3 or OKT3 + B7-1 Fc for 72 h. CFSE dilution was measured with flow cytometry. Bar plot summarizes data from three independent experiments. **e** Naive CD4 T cells were isolated and stimulated with OKT3, OKT3 + Ctrl Fc, OKT3 + B7-1 Fc, and OKT3 + B7-H7 Fc for 48 h. Cell cycle analysis was performed by staining cells with EdU. Percentage of cells in G, S, and G2 phase was calculated with Flowjo software. **f** Total CD4 T cells were stimulated with OKT3 alone or OKT3 + B7-H7 Fc, and pERK was measured with flow analysis. MFI values are summarized in the table for each treatment group. This experiment was repeated with three different donors (histograms in gray: unstained, blue: unstimulated, green: OKT3, and orange: OKT3 + B7-H7 Fc) (***p ≤ 0.0001, ***p ≤ 0.001, **p ≤ 0.005, *p ≤ 0.05)

Although B7-H7 was not as potent as B7-1 in inducing activation genes, there was still a significant difference compared with OKT3 alone. We next tested whether mRNA induction translated into protein expression on the cell surface. Purified naive CD4 T cells were stimulated with OKT3 only or in combination with a control Fc (Ctrl Fc), B7-1 Fc, or B7-H7 Fc chimeric protein for 72 h. Although *CD69* and *CD25* were upregulated at the mRNA level in the presence of B7-H7, as shown in Fig. 1d, these increases did not translate into protein expression on the cell surface, as demonstrated by flow cytometry (Fig. 2a). Cytokine levels in supernatants were also measured. OKT3 and B7-1 treatment of T cells resulted in a robust and significant induction of IL-2 and IFN-γ, whereas OKT3 and B7-H7 treatment did not induce any cytokines (Fig. 2b). CFSE (Carboxyfluorescein succinimidyl ester) dilution studies were performed to evaluate the impact of B7-H7 on T-cell proliferation. B7-1 stimulation of naive CD4 T cells resulted in robust proliferation of the cells, whereas B7-H7 treatment failed to drive cell division in the context of OKT3 (Fig. 2c). The impact of OKT3 and B7-H7 on additional T-cell populations was also studied, including naive CD8 T cells and memory CD4 T cells. OKT3 and B7-H7 did not induce proliferation in any of the cell populations tested (Supplementary Fig. 2a).

We further explored the underlying basis for the inability of B7-H7 to provide costimulation in combination with OKT3. Towards this goal, the impact of pre-exposure to B7-H7 on the responsiveness of T cells was studied. Naive T cells were exposed to OKT3 and B7-H7 Fc overnight, washed, and cultured in the presence of plate-bound OKT3 and B7-1 Fc. The T cells that were pretreated with OKT3 and B7-H7 failed to proliferate in response to subsequent OKT3 and B7-1 stimulation, whereas the cells that were pre-exposed to OKT3 and Ctrl Fc proliferated effectively (Fig. 2d). Cell cycle analysis was also performed after OKT3 and B7-H7 treatment, and cells were stained with EdU and cultured with plate-bound reagents for 24 h. During that time, ~50% of the cells incorporated EdU, as they divided in B7-1-treated conditions, whereas none of the cells entered the cell cycle after B7-H7 treatment (Fig. 2e). To examine intracellular signaling pathways induced by B7-H7, pERK protein levels after B7-H7 treatment were measured. Interestingly, B7-H7 reduced OKT3-induced pERK levels, suggesting that B7-H7 can inhibit OKT3 signaling (Fig. 2f).

Engagement of TCR with distinct α-CD3 clones has been shown to differentially impact T-cell activation; therefore, we evaluated the influence of B7-H7 signals in combination with a second α-CD3 clone, UCHT1. Surprisingly, B7-H7 induced CD25, CD69, and PD-1 protein expression on T cells when combined with UCHT1 (Supplementary Fig. 3a). In addition, supernatants were collected and the IL-2 and IFN-γ levels were measured. B7-1 treatment of T cells resulted in a robust and significant induction of cytokine production in the presence of both OKT3 and UCHT1. It is important to note that the cytokine levels were much higher in OKT3-treated cells than in cells treated with UCHT1 in combination with B7-1. B7-H7 treatment in combination with OKT3 did not induce either cytokine; however, in combination with UCHT1, B7-H7 significantly augmented IFN-γ production (Supplementary Fig. 3b). Significant proliferation was observed when naive T cells were stimulated with UCHT1 and B7-H7, although the greatest degree of proliferation was observed with UCHT1 and B7-1 (Supplementary Fig. 3c). The implications of these differential functional outcomes are discussed in detail in the Discussion section.

### B7-H7 inhibits T cells in the presence of TCR and CD28 signaling

**Fig. 3:**
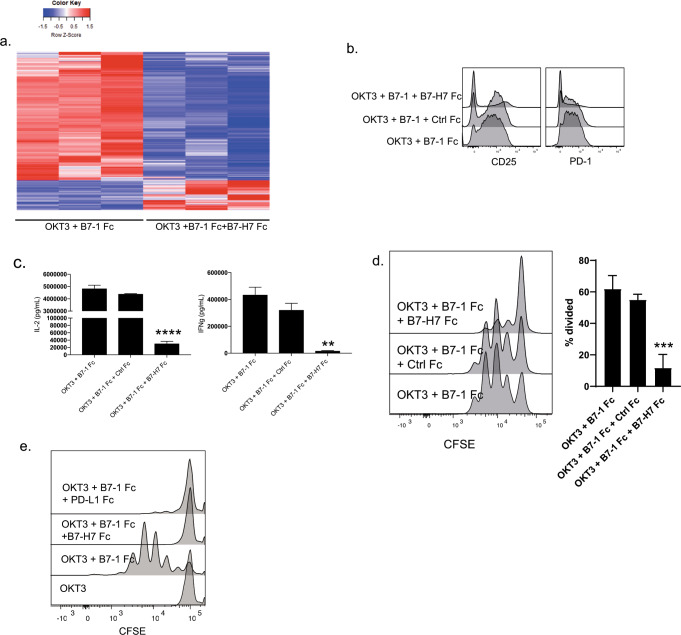
B7-H7 inhibits T cells in the presence of TCR and CD28 signaling. **a** Naive CD4 T cells were purified and stimulated with OKT3 + B7-1 Fc, OKT3 + B7-1 Fc + Ctrl Fc, and OKT3 + B7-1 Fc + B7-H7 Fc for 2 h. RNA was isolated and whole-transcriptome sequencing was performed. Heatmap of genes that are significantly regulated in RNA-sequencing studies. OKT3 + B7-1 Fc-treated cells were used as the reference sample in the generation of the heatmap (*n* = 3 donors, fold change >4, FDR <0.25). **b** CD25 and PD-1 protein expression on naive CD4 T cells after 72 h stimulation under indicated conditions. **c** Cell culture supernatants were collected at 72 h, and IL-2 and IFNγ were measured. **d** CFSE dilution was measured in the presence of plate-bound OKT3 + B7-1 Fc, OKT3 + B7-1 Fc + Ctrl Fc, and OKT3 + B7-1 Fc + B7-H7 Fc. Bar plot summarizes data from three independent experiments. **e** Naive CD4 T cells were stimulated with beads coated with indicated reagents and proliferation was measured 72 h later by flow cytometry. (***p ≤ 0.0001, ***p ≤ 0.001, **p ≤ 0.005, *p ≤ 0.05)

Next, we studied the outcomes of T cells receiving B7-H7 signaling in the presence of both TCR and CD28 signaling. RNAseq studies demonstrated that stimulation of naive CD4 T cells with OKT3, B7-1, and B7-H7 resulted in changes to the global transcriptome compared with OKT3 and B7-1 stimulation (Fig. 3a). The genes that were turned off in the OKT3 and B7-1 group were turned on in the OKT3, B7-1, and B7-H7 group and vice versa. The inhibition of mRNA induction was also reflected in protein expression on the cell surface, as evidenced by the CD25 and PD-1 expression results (Fig. 3b). The addition of Ctrl Fc to OKT3 and B7-1 did not downregulate activation marker expression, but simultaneous stimulation with B7-H7 did. This coinhibitory effect on activation marker expression was also evident to a lesser degree in the presence of UCHT1, B7-1, and B7-H7 (Supplementary Fig. 3d). Cytokine levels in supernatants were also measured and B7-H7 significantly reduced IL-2 and IFNγ production in the presence of OKT3 (Fig. 3c) and only IFNγ production in combination with UCHT1 (Supplementary Fig. 3e). As mentioned before, T cells proliferated robustly in the presence of OKT3 and B7-1; however, simultaneous stimulation with B7-H7 significantly reduced proliferation (Fig. 3d). Coinhibition with B7-H7 still occurred with UCHT1 and B7-1, but was not as dramatic as that observed with the presence of OKT3 (Supplementary Fig. 3f).

We also compared the inhibitory abilities of B7-H7 and PD-L1. For these experiments, bead-bound reagents were used, as this approach has been shown to be the most effective method of PD-L1 delivery^23^ PD-L1 needs to be delivered in cis with OKT3 and usually the ratio of PD-L1 to OKT3 needs to be high.^24^ In this case, 4:1 PD-L1:OKT3 or B7-H7:OKT3 was used, and our data showed that B7-H7 and PD-L1 were equally effective in reducing T-cell inhibition (Fig. 3e).

### Blocking the B7-H7 and CD28H interaction increases T-cell proliferation in mixed lymphocyte reaction assays

**Fig. 4:**
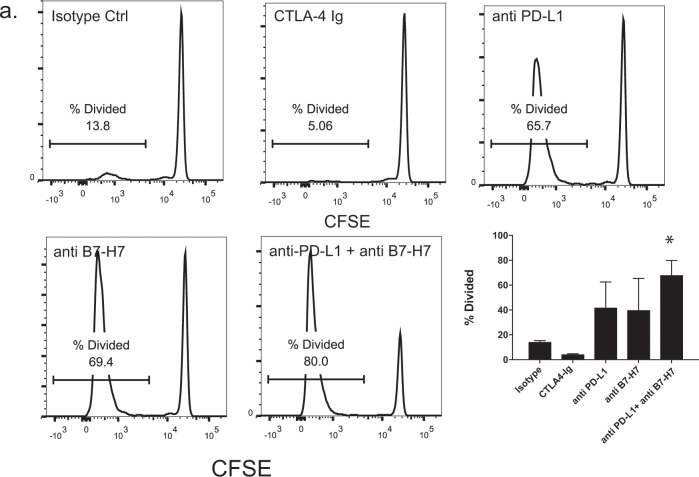
Blocking B7-H7 and CD28H interaction increases T-cell proliferation and activation in physiological setting. **a** Naive CD4 T cells were isolated from one donor, CFSE-labeled, and placed in co-culture with purified DCs from another donor. The cells were incubated for 7 days in the presence of isotype control, CTLA-4-Ig, α-PD-L1, α-B7-H7, or anti-PD-L1 + α-B7-H7, and CFSE dilution was measured. All antibodies were used at 10 µg/ml. Percent divided cells were quantified from three different experiments (CFSE plots are representative of one of three donors tested, *p ≤ 0.05)

So far, these studies have used an antibody (specific for CD3) and a chimeric protein (for other ligands) to stimulate T cells without any antigen-presenting cells. We wanted to test the activity of B7-H7 in the context of antigen-presenting cells. To this end, mixed lymphocyte reactions were set up by using purified naive T cells from one donor and purified DCs from another donor. The cells were mixed at a 1:2 DC to T-cell ratio and incubated for 7–10 days in the presence of different antibodies. CTLA-4 Ig was used as a control for T-cell inhibition and an α-PD-L1 antibody was used as a control for T-cell activation. Blockade of B7-H7 with a specific monoclonal antibody resulted in increased T-cell proliferation. When α-PD-L1 and anti-B7-H7 antibodies were simultaneously used for blocking, there was an additive effect on T-cell proliferation (Fig. 4a).

## Discussion

The role of the B7-H7 and CD28H pathway in T-cell activation has been controversial. Further understanding of this pathway would be beneficial in the generation of new therapeutics for modulating T-cell function. To this end, we systematically studied the effects of B7-H7 in combination with TCR and CD28 signaling. Our study is the first to examine the transcriptomic profile of naive T cells after B7-H7 stimulation. OKT3 and B7-H7 stimulation upregulated the expression of ~47% of the genes that also exhibited upregulated expression induced by B7-1; however, some key genes were not induced at all with B7-H7. For the genes that exhibited upregulated expression induced by both B7-1 and B7-H7, the fold changes were much lower with B7-H7, suggesting that there may be a threshold for proper activation. The expression of 441 genes was uniquely upregulated by B7-1, and without the induction of those genes, T cells are unable to produce cytokines and proliferate effectively. Only ~12% of the genes with expression downregulated by B7-1 treatment also were downregulated by B7-H7, suggesting that these two molecules may engage different pathways. In the list of genes with expression uniquely upregulated by B7-H7, some extracellular matrix (ECM) interaction genes were evident (i.e., *COL6A3, LIMA1, and VASP*). B7-H7 has been previously demonstrated to be important in the metastasis of human osteosarcoma and one study hypothesized that cancer cells may upregulate this ligand to form metastatic colonies in new tissues, providing evidence for the importance of B7-H7 in cell–cell adhesion.^22^ CD28H has also been demonstrated to be a novel adhesion molecule involved in angiogenesis and the expression of this receptor on epithelial and endothelial cells regulates cell–cell interactions, cell migration, and angiogenesis within tissues.^25^ It has also been demonstrated that CD28H expression is upregulated on primary colon cancer cells and this upregulation results in increased cell–cell adhesion, tumor cell aggregation, and tumor growth promotion.^26^ Our transcriptomic data suggest that B7-H7 may also regulate cell–cell adhesion or ECM interactions in primary human T cells. Further studies are required to examine the role of B7-H7 in T-cell adhesion to the ECM.

Following careful examination of transcriptomic changes, the biological effects of T-cell stimulation with B7-H7 in the presence of TCR signaling were studied. Previous studies have differences in experimental design and the reagents used, which may explain some of their contradictory outcomes. For example, Zhao et al.^15^ used total T cells in all of their studies without further separation of CD4 vs. CD8 or naive vs. memory T cells. As they used total T cells, they observed basal proliferation and cytokine production with an α-CD3 antibody (OKT3) and demonstrated that the addition of B7-H7 was coinhibitory. When purified naive CD4 cells were stimulated with OKT3, we did not observe activation marker expression, cytokine production, or proliferation, and in this case, B7-H7 did not deliver costimulation. A proper control (Control Fc) was used in addition to OKT3 and the addition of B7-H7 did not result in additional cytokine production or proliferation compared with the control alone. Zhu et al.^16^ used naive or total CD4 or CD8 T cells depending on the experiment and, in most cases, used anti-CD28H antibodies for proliferation and cytokine production studies. This antibody strategy may be the main differentiating factor for the stimulatory effects that they observed. There are no studies closely examining the binding characteristics of B7-H7 vs. α-CD28H antibodies; however, if they engage CD28H differently, this may also explain the differences downstream of the receptor.

We wanted to mechanistically understand the lack of costimulation in the presence of OKT3 and B7-H7. Pretreatment studies demonstrated that naive T cells became unresponsive to OKT3 and B7-1 stimulation after being treated with B7-H7 overnight. Previously, T-cell anergy in the presence of co-inhibitors such as PD-L1 and in the tumor microenvironment has been studied. Anergy induction is proposed to be one of the mechanisms of immune evasion. The T-cell unresponsiveness that we observed after B7-H7 treatment may also be another immune escape mechanism that is employed by tumor types that express B7-H7.^27^ In addition, T cells were unable to enter the cell cycle after OKT3 and B7-H7 stimulation. One reason for this could be that the phosphorylation of ERK was markedly reduced with the addition of B7-H7 when compared with OKT3 stimulation alone. Zhu et al.^16^ previously reported that B7-H7 stimulation resulted in the phosphorylation of Akt. In their study, the type of T cells used was unclear and whether the treatment was administered in combination with OKT3 was not indicated. Nonetheless, we were not able to detect reproducible results for pAkt.

Different α-CD3 clones may have differential effects on T-cell function and, to address this question, UCHT1 was used as an alternate clone. Although OKT3 and UCHT1 are specific for overlapping epitopes in CD3ε, different biological outcomes have been demonstrated in terms of functionality. Indeed, according to the Tunnacliffe classification system, OKT3 and UCHT1 belong to different subgroups of anti-CD3 clones. OKT3 recognizes conformational determinants that rely on the binding of CD3ε to other subunits and UCHT1 recognizes the native CD3ε-TCR complex.^28^ In terms of functional effects, it has been shown that OKT3 activates T cells in an IL-2-dependent manner, whereas UCHT1 activity is IL-2 independent.^29^ In our studies, the lack of IL-2 production when T cells were stimulated with UCHT1 and B7-H7 was an indication that the T cells were activated in an IL-2-independent manner in this setting as well. In a separate study, the capacity of different α-CD3 clones to induce proliferation in the absence of costimulatory signals was studied. This paper demonstrated that suboptimal concentrations of OKT3 induced proliferation only when IL-1β was added as an accessory signal, while stimulation with the same concentration of UCHT1 resulted in T-cell proliferation in the absence of a second signal.^30^ Interestingly, a recent paper exploring new ways to generate less toxic chimeric antigen receptors came up with a new receptor format that they named T-cell antigen coupler (TAC). TAC has three components: an antigen-binding domain, a TCR recruitment domain, and a coreceptor domain. The authors hypothesized that this format would activate T cells in a more controlled manner without costimulation, leading to reduced toxicity. Their studies demonstrated that the use of OKT3 in this setting produced poor proliferation and cytokine production, and that UCHT1 TACs demonstrated superior performance in preclinical models of solid and hematological tumors.^31^ In our study, we demonstrate that B7-H7 cotreatment with OKT3 has opposing outcomes when compared with cotreatment with UCHT1, which could be explained by discrepancies in epitope specificity and the strength of the signal delivered by the different α-CD3 clones. In addition, as discussed later, we believe that OKT3 is the superior clone that should be used in these assays, as it reflects the biological outcomes of in vitro physiological assays.

T cells integrate many signals at once; therefore, we wanted to test the biological outcome of B7-H7 stimulation in the presence of TCR and CD28 signaling. Previously, Zhu et al.^16^ stimulated T cells with α-CD28 and α-CD28H antibodies, and demonstrated a synergistic effect on T-cell proliferation. Our study is the first to use endogenous ligands simultaneously and we show the coinhibitory effect of B7-H7 with OKT3 and B7-1, and to a lesser degree that of B7-H7 with UCHT1 and B7-1. In the experiments that used UCHT1 as signal 1, it is possible that B7-1 and B7-H7 blocked each other; however, more studies are required to demonstrate this interaction. Global transcriptomic data show the striking effect of B7-H7 on B7-1-induced genes; however, we are not able to determine whether B7-H7 actively turns off B7-1 downstream signaling or whether it simply does not allow effective B7-1 signaling. In addition, we are not able to conclude whether the coinhibitory activity of B7-H7 is CD28H dependent. Further studies are needed to address these issues. The coinhibitory effect of B7-H7 was also evident in the reduction in activation marker expression and T-cell proliferation. One of the most interesting pieces of data we obtained suggested that B7-H7 may be as effective as PD-L1 in inhibiting T-cell proliferation in vitro, as demonstrated by bead proliferation experiments. In support of our data, a recent study of a cohort of non-small cell lung carcinoma patients demonstrated that PD-L1 expression was shown in 25% and 31% of tumors in the discovery and validation cohorts, respectively, whereas B7-H7 expression was observed in 61% and 64% of tumors, respectively. This study also demonstrated that B7-H7 Ig inhibited CD4 and CD8 T-cell proliferation more significantly than did PD-L1 Ig.^21^

Stimulation of T cells via their TCR and costimulatory receptors is effective yet may not reflect the true nature of the MHC–TCR interaction. To mimic this interaction more closely, mixed lymphocyte reaction experiments were used. In this setting, in the presence of APC and T-cell interactions, blocking B7-H7 produced increased proliferation and activation of T cells. As shown previously, activated DCs express B7-H7 and B7-H7 acts as a “break” signal to suppress activation. Furthermore, transcriptomic and functional data generated with OKT3 mirror the data obtained from these physiological studies; therefore, we strongly believe that B7-H7 is an inhibitory ligand.

**Fig. 5:**
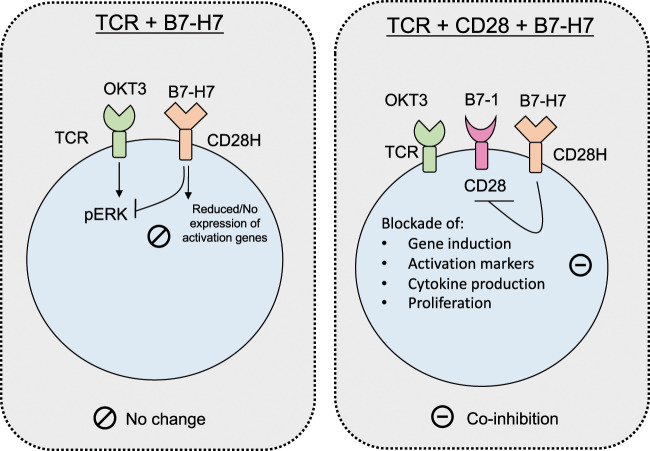
Biological outcomes of B7-H7 exposure during TCR and CD28 signaling. When combined with OKT3, B7-H7 does not provide costimulation due to suboptimal induction of activation genes, reduction in pERK, inability to enter cell cycle, and induction of unresponsive state. In the presence of TCR and CD28 signaling, B7-H7 is coinhibitory and blocks activation, cytokine production, and proliferation of T cells

In conclusion, B7-H7 stimulation results in suboptimal transcriptomic changes in T cells with no functional outcomes in terms of activation, cytokine production, and proliferation. These findings suggest that there is a threshold for gene activation before T cells can be fully activated. Furthermore, B7-H7 is inhibitory only in the presence of both TCR and CD28 signaling in T cells, suggesting that the inhibitory effects are CD28 dependent (Fig. 5a). Detailed studies are needed to identify the intracellular players in the downstream inhibitory pathways.

## Materials and methods

### PBMC isolation and T-cell purification

All experiments using human blood were approved by and carried out in accordance with the Advatta Institutional Review Board. All experimental protocols were approved by the MedImmune Blood Donor Program. Written consent was obtained from healthy adult volunteers who were employees of MedImmune or AstraZeneca, and samples were anonymized for research purposes. Donors positive for HIV infection, hepatitis B or C virus infection, human T-lymphotropic virus infection, or syphilis were excluded from the donor program. Whole blood from healthy volunteers was collected in heparinized CPT tubes. The tubes were spun at 1700 × *g* for 20 min at room temperature. The supernatant above the plug was collected into new tubes and spun at 300 × *g* for 20 min with the brake off. The supernatant was discarded, and the PBMC pellet was resuspended in RoboSep buffer (StemCell Technologies, catalog number 20104). The cells were then separated according to the manufacturer’s instructions using negative selection kits for naive CD4 T cells (StemCell Technologies, catalog number 19155), naive CD8 T cells (StemCell Technologies, catalog number 19158), memory CD4 T cells (StemCell Technologies, catalog number 19157), and memory CD8 T cells (StemCell Technologies, catalog number 19159).

### CFSE labeling of T cells

After purification, T cells were spun at 300 × *g* for 5 min at room temperature, resuspended in phosphate-buffered saline (PBS), and labeled using a Cell Trace CFSE cell proliferation kit (Invitrogen, catalog number C34554). Briefly, reconstituted CFSE was added at a 1:2000 dilution for 8 min in a 37 °C water bath. Immediately after, an equivalent volume of fetal bovine serum was added to stop the labeling reaction and the cells were incubated for an additional 5 min at room temperature. The T cells were then washed with X-Vivo 15 medium (Lonza, catalog number 04-418Q), resuspended, and counted for further experiments.

### Measurement of T-cell activation and proliferation

T cells were plated at 2.5 × 10^5^/well/ml in precoated 24-well plates (non-TC treated; Costar, catalog number 3738). For plate coating, the following reagents were used: anti-CD3 OKT3 (BioLegend, catalog number 317326; 1 mg/ml), anti-CD3 UCHT1 (BD Biosciences, catalog number 555329), IgG control Fc chimera (R&D, 110-HG), B7.1 Fc chimera (R&D, 140-B1; 1 mg/ml), and B7-H7 Fc chimera (R&D, 8084-B7-050; 1 mg/ml). We used OKT3 and UCHT1 at 1 µg/ml and the IgG Control Fc, B7.1 Fc, and B7-H7 Fc at 5 µg/ml (unless otherwise indicated). The plates were incubated at 37 °C for 2 h and washed with PBS twice, and cells were plated for 72 h at 37 °C.

In some experiments, precoated Dynabeads beads were used to stimulate T cells. A Dynabead-antibody coupling kit (Life Technologies, catalog number 14311D) was used to attach OKT3, Ctrl Fc, B7-1 Fc, B7-H7 Fc, or PD-L1 Fc to beads according to the manufacturer’s instructions. A 1:4 ratio of anti-CD3 antibody to B7-1, which was determined to be optimal for T-cell stimulation, was used for coating the beads (40 µg of OKT3 and 160 µg of the ligands) unless otherwise indicated in the figure. The total amount of antibody used to coat the beads was 200 µg/ml of beads.

After T-cell stimulation, cells were collected and washed with PBS. Next, the cells were stained for flow cytometric analysis with the following reagents for 30 min at 4 °C: Live/Dead dye and anti-TCRαβ, anti-CD4, anti-CD8, anti-CD25, anti-CD69, and anti-PD-1 antibodies. The cells were then washed with PBS twice and analyzed with an LSR flow cytometer.

### Measurement of cytokine levels

After T cells were activated for 72 h, the supernatants were collected. MsD (Meso Scale Discovery) analysis was performed to measure IL-2, IFN-γ, tumor necrosis factor-α, and IL-6 levels according to the manufacturer’s instructions.

### Gene expression analysis by RT-PCR

For reverse-transcriptase PCR (RT-PCR) studies, T cells were activated in the absence or presence of different treatment conditions for 2 h. The cells were collected and washed twice with PBS and RNA was isolated using a Qiagen Shredder and RNA isolation kit. After measurement of the RNA concentration with a Nanodrop, cDNA synthesis was performed with an x kit and RT-PCR analysis was carried out with the following probes from TaqMan assays: IL-2, IFNγ, NFKBIA, and PD-1.

### RNAseq studies

RNAseq studies were performed by Covance. The quality of the RNA was assessed based on the RNA profile generated by a bioanalyzer using a nanochip. Removal of rRNA and globin-encoding mRNA, RNA fragmentation, cDNA generation, adapter ligation, and PCR amplification were performed using a TruSeq stranded total RNA with ribo-zero globin sample preparation kit (Illumina) and 50 bp paired-end sequencing was performed using an Illumina HiSeq4000.

### pERK level measurement

After a Ficoll (Corning™ cellgro™ Lymphocyte Separation Medium) purification step (as recommended by the manufacturer), PBMCs were stimulated using 1 × 10^6^ cells for each condition. For soluble stimulation, the following reagents were used: anti-CD3 OKT3 (BioLegend, catalog number 317326; 4 mg/ml) and B7-H7 Fc chimera (R&D, 8084-B7-050; 1 mg/ml). We used OKT3 and B7-H7 Fc at 5 µg/ml. Cells were incubated at 37 °C for 5 min. After PBMC stimulation, the cells were collected and washed twice with PBS/2% fetal calf serum (FCS; Thermo Fisher). We blocked Fc receptors by incubating for 10 min in PBS/10% FCS at room temperature. Next, the cells were stained with an anti-CD3 antibody (PerCP-Cy™5.5 Mouse Anti-Human CD3, BD) for 30 min at 4 °C in PBS/2% FCS. The cells were then washed with PBS-0.5% bovine serum albumin (BSA; Fisher Scientific) twice and fixed for 15 min at room temperature with 2% paraformaldehyde (Alfa Aesar™ Paraformaldehyde, 97%, Alfa Aesar). Permeabilization was performed with PBS/0.5% BSA/0.1% saponin (saponin, Sigma). Intracellular staining was performed with a 1:1000 dilution of an anti-p-ERK antibody (Cell Signaling) and the cells were washed twice with the PBS/BSA/saponin solution. An Alexa Fluor 488-conjugated goat anti-mouse IgG secondary antibody (Invitrogen) was used at a 1:1000 dilution and the cells were washed twice with PBS/BSA/saponin, washed again with the PBS/2% FCS (Thermo Fisher) solution, and analyzed with an LSRII flow cytometer.

### Mixed lymphocyte reaction

Naive CD4 T cells were isolated from one donor as described above using RoboSep separation. The T cells were CFSE labeled as described above. Dendritic cells were isolated from another donor’s PBMCs by using a pan-DC separation kit (StemCell Technologies, catalog number 19251). The DCs and T cells were mixed at a 1:2 ratio in 96-well U-bottom plates and cultured for 7–10 days at 37 °C. The DCs and T cells were either cultured without any treatment or in the presence of an isotype control, CTLA-4-Ig (Abatacept), α-PD-L1 (BioLegend, catalog number 329715), or α-B7-H7 (Amplimmune, Clone 20C5). All of the antibodies were used at 10 µg/ml. After the culture period, CFSE dilution was measured by flow cytometry as a read out for cell proliferation.

## Supplementary information

Supplementary Legend

Supplementary Figures

## References

[1] Ai M, Curran MA (2015). Immune checkpoint combinations from mouse to man. Cancer Immunol. Immunother..

[2] Rosman Z, Shoenfeld Y, Zandman-Goddard G (2013). Biologic therapy for autoimmune diseases: an update. BMC Med..

[3] Sharma P, Allison JP (2015). The future of immune checkpoint therapy. Science.

[4] Getts DR (2011). Current landscape for T-cell targeting in autoimmunity and transplantation. Immunotherapy.

[5] Kamta J (2017). Advancing cancer therapy with present and emerging immuno-oncology approaches. Front. Oncol..

[6] Alsaab HO (2017). PD-1 and PD-L1 checkpoint signaling inhibition for cancer immunotherapy: mechanism, combinations, and clinical outcome. Front. Pharm..

[7] Najafian N, Sayegh MH (2000). CTLA4-Ig: a novel immunosuppressive agent. Expert Opin. Investig. Drugs.

[8] Brownlie RJ, Zamoyska R (2013). T cell receptor signalling networks: branched, diversified and bounded. Nat. Rev. Immunol..

[9] Beyersdorf N, Kerkau T, Hunig T (2015). CD28 co-stimulation in T-cell homeostasis: a recent perspective. Immunotargets Ther..

[10] Lauvau G, Soudja SM (2015). Mechanisms of memory T cell activation and effective immunity. Adv. Exp. Med. Biol..

[11] Murakami N, Riella LV (2014). Co-inhibitory pathways and their importance in immune regulation. Transplantation.

[12] Ceeraz S, Nowak EC, Noelle RJ (2013). B7 family checkpoint regulators in immune regulation and disease. Trends Immunol..

[13] Chen L, Flies DB (2013). Molecular mechanisms of T cell co-stimulation and co-inhibition. Nat. Rev. Immunol..

[14] Jung K, Choi I (2013). Emerging co-signaling networks in T cell immune regulation. Immune Netw..

[15] Zhao R (2013). HHLA2 is a member of the B7 family and inhibits human CD4 and CD8 T-cell function. Proc. Natl Acad. Sci. USA.

[16] Zhu Y (2013). B7-H5 costimulates human T cells via CD28H. Nat. Commun..

[17] Crespo J (2017). Phenotype and tissue distribution of CD28H(+) immune cell subsets. Oncoimmunology.

[18] Zhuang X, Long EO (2019). CD28 homolog is a strong activator of natural killer cells for lysis of B7H7(+) tumor cells. Cancer Immunol. Res.

[19] Janakiram M (2015). Expression, clinical significance, and receptor identification of the newest B7 family member HHLA2 protein. Clin. Cancer Res..

[20] Xiao Y, Freeman GJ (2015). A new B7:CD28 family checkpoint target for cancer immunotherapy: HHLA2. Clin. Cancer Res..

[21] Cheng H (2018). Wide expression and significance of alternative immune checkpoint molecules, B7x and HHLA2, in PD-L1-negative human lung cancers. Clin. Cancer Res..

[22] Koirala P (2016). HHLA2, a member of the B7 family, is expressed in human osteosarcoma and is associated with metastases and worse survival. Sci. Rep..

[23] Bennett F (2003). Program death-1 engagement upon TCR activation has distinct effects on costimulation and cytokine-driven proliferation: attenuation of ICOS, IL-4, and IL-21, but not CD28, IL-7, and IL-15 responses. J. Immunol..

[24] Butte MJ (2007). Programmed death-1 ligand 1 interacts specifically with the B7-1 costimulatory molecule to inhibit T cell responses. Immunity.

[25] Rahimi N (2012). Identification of IGPR-1 as a novel adhesion molecule involved in angiogenesis. Mol. Biol. Cell.

[26] Woolf N (2017). Targeting tumor multicellular aggregation through IGPR-1 inhibits colon cancer growth and improves chemotherapy. Oncogenesis.

[27] Crespo J (2013). T cell anergy, exhaustion, senescence, and stemness in the tumor microenvironment. Curr. Opin. Immunol..

[28] Tunnacliffe A, Olsson C, de la Hera A (1989). The majority of human CD3 epitopes are conferred by the epsilon chain. Int. Immunol..

[29] Van Wauwe JP, Goossens JG, Beverley PC (1984). Human T lymphocyte activation by monoclonal antibodies; OKT3, but not UCHT1, triggers mitogenesis via an interleukin 2-dependent mechanism. J. Immunol..

[30] Verwilghen J (1991). Differences in the stimulating capacity of immobilized anti-CD3 monoclonal antibodies: variable dependence on interleukin-1 as a helper signal for T-cell activation. Immunology.

[31] Helsen CW (2018). The chimeric TAC receptor co-opts the T cell receptor yielding robust anti-tumor activity without toxicity. Nat. Commun..

