# Supplementary material for: B7-H7 (HHLA2) inhibits T-cell activation and proliferation in the presence of TCR and CD28 signaling
Source: Cell Mol Immunol. 2020 Jan 31;18(6):1503–11. doi: 10.1038/s41423-020-0361-7 (PMC8166953; doi:10.1038/s41423-020-0361-7)
Supplement: Supplementary file 1 — Supplementary Legend [file 41423_2020_361_MOESM1_ESM.docx]

**Supplementary Figure 1.** Naïve CD4 T cells were isolated and stimulated with different treatment groups for 2 hours. RNA was isolated and RT-PCR for IL-2, IFNg and EGR1 was carried out. (b) PCA clustering of different treatment groups from RNA sequencing, n=3 donors. Different colors represent different donors.

**Supplementary Figure 2.** Naïve CD8 and memory T cells were isolated and stimulated with OKT3, OKT3 and B7-1, OKT3 and B7-H7 and CFSE dilution was measured 72 hours later.

**Supplementary Figure 3**. (a) Naïve CD4 T cells were stimulated with UCHT1 and in combination with chimera proteins for 72 hours. The cells were then stained for CD25, CD69 and PD-1 protein expression, (b) IL-2 and IFNγ were measured in cell culture supernatants and (c) CFSE dilution was measured for cell proliferation in the presence of UCHT1. Naïve CD4 T cells were stimulated with UCHT1 and B7-1 and combined with Ctrl Fc or B7-H7 Fc. (d) Activation markers, (e) cytokine production and (f) proliferation was measured 72hrs after stimulation.
