# Supplementary figures and images for: B7-H7 (HHLA2) inhibits T-cell activation and proliferation in the presence of TCR and CD28 signaling
Source: Cell Mol Immunol. 2020 Jan 31;18(6):1503–11. doi: 10.1038/s41423-020-0361-7 (PMC8166953; doi:10.1038/s41423-020-0361-7)

Supplementary Figure 1

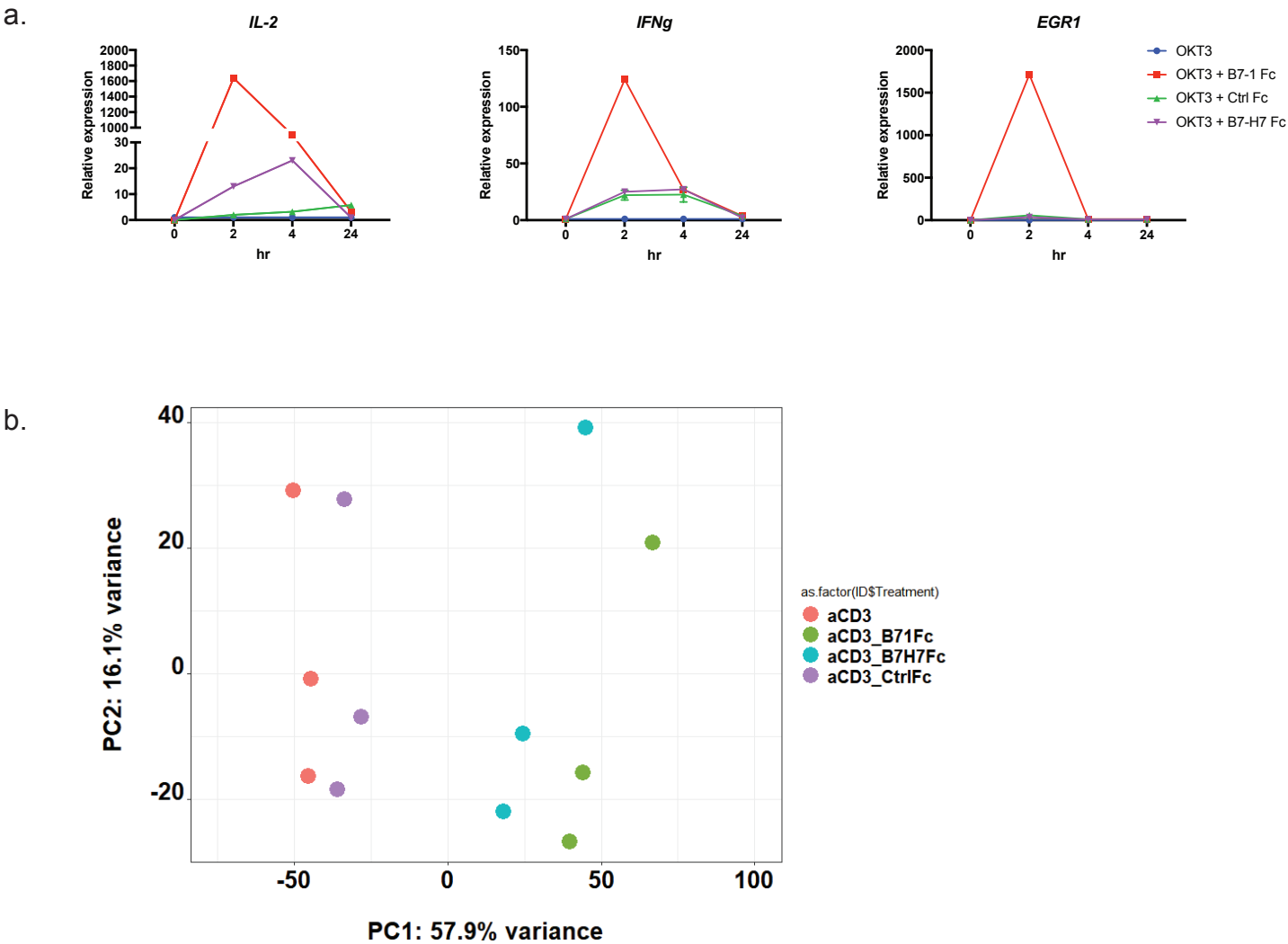

Supplementary Figure 2

a.

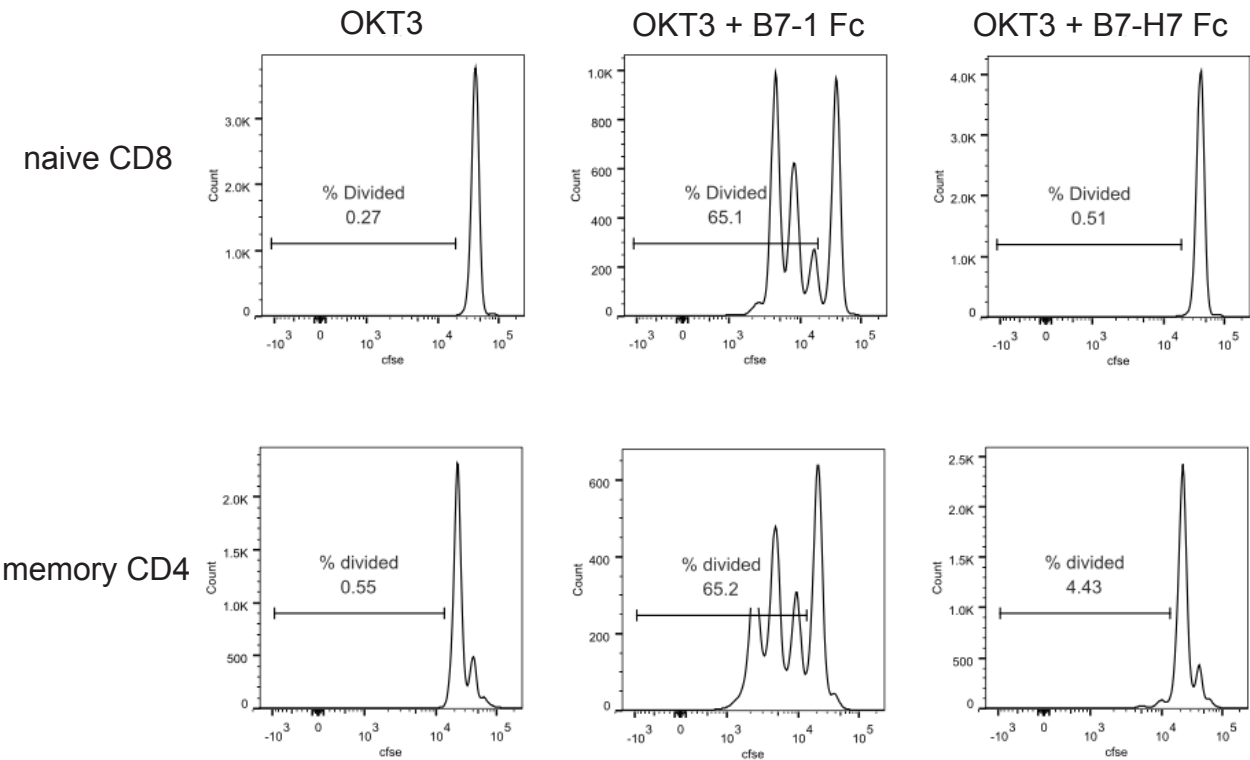

Supplemental Figure 3

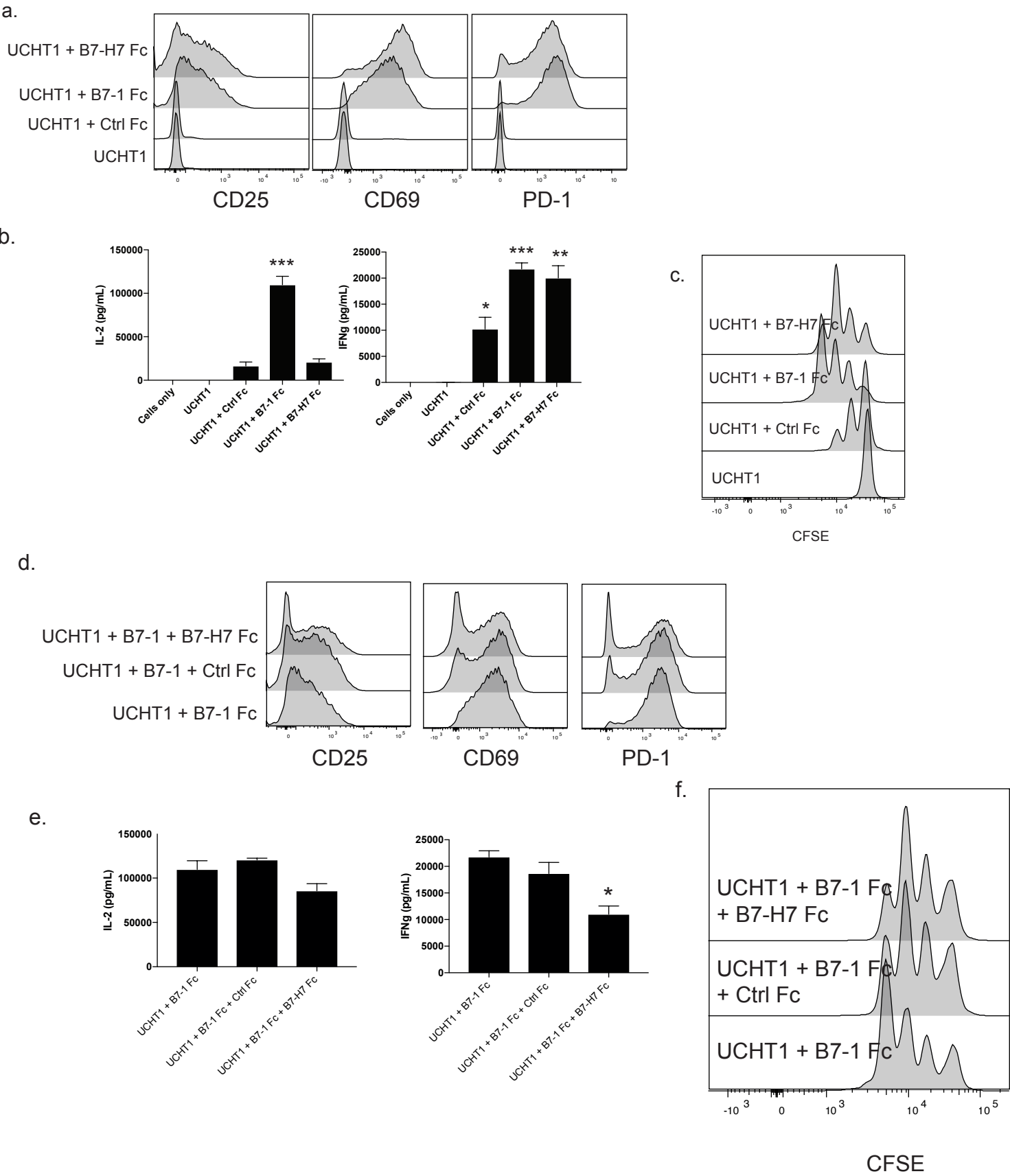

Supplement: Supplementary file 2 — Supplementary Figures [file 41423_2020_361_MOESM2_ESM.pdf]
